# Inter Subject Variability and Reproducibility of Diffusion Tensor Imaging within and between Different Imaging Sessions

**DOI:** 10.1371/journal.pone.0065941

**Published:** 2013-06-28

**Authors:** Tonny V. Veenith, Eleanor Carter, Julia Grossac, Virginia F. J. Newcombe, Joanne G. Outtrim, Victoria Lupson, Guy B. Williams, David K. Menon, Jonathan P. Coles

**Affiliations:** 1 Division of Anaesthesia, University of Cambridge, Cambridge, Cambridgeshire, United Kingdom; 2 Wolfson Brain Imaging Centre, Addenbrooke's Hospital, Cambridge, Cambridgeshire, United Kingdom; Institute of Psychology, Chinese Academy of Sciences, China

## Abstract

The aim of these studies was to provide reference data on intersubject variability and reproducibility of diffusion tensor imaging. Healthy volunteers underwent imaging on two occasions using the same 3T Siemens Verio magnetic resonance scanner. At each session two identical diffusion tensor sequences were obtained along with standard structural imaging. Fractional anisotropy, apparent diffusion coefficient, axial and radial diffusivity maps were created and regions of interest applied in normalised space. The baseline data from all 26 volunteers were used to calculate the intersubject variability, while within session and between session reproducibility were calculated from all the available data. The reproducibility of measurements were used to calculate the overall and within session 95% prediction interval for zero change. The within and between session reproducibility data were lower than the values for intersubject variability, and were different across the brain. The regional mean (range) coefficient of variation figures for within session reproducibility were 2.1 (0.9–5.5%), 1.2 (0.4–3.9%), 1.2 (0.4–3.8%) and 1.8 (0.4–4.3%) for fractional anisotropy, apparent diffusion coefficient, axial and radial diffusivity, and were lower than between session reproducibility measurements (2.4 (1.1–5.9%), 1.9 (0.7–5.7%), 1.7 (0.7–4.7%) and 2.4 (0.9–5.8%); p<0.001). The calculated overall and within session 95% prediction intervals for zero change were similar. This study provides additional reference data concerning intersubject variability and reproducibility of diffusion tensor imaging conducted within the same imaging session and different imaging sessions. These data can be utilised in interventional studies to quantify change within a single imaging session, or to assess the significance of change in longitudinal studies of brain injury and disease.

## Introduction

Diffusion tensor imaging (DTI) has been used to identify neuronal injury and predict outcome in a variety of neurological disorders such as traumatic brain injury [1]–[3], multiple sclerosis [4]–[6], Alzheimer's dementia and psychiatric disorders [7]–[9]. Previous human studies using DTI have provided invaluable reference data regarding normal values within different brain structures and several groups have reported data comparing DTI measurements between subjects, between scanners in different centres, following service upgrades, and reproducibility within the same centre over time [10]–[28]. However, there are limited data that compare intersubject variability and reproducibility of DTI measurements [23], or published studies that compare reproducibility of DTI measurements obtained within the same imaging session (within session reproducibility) with that obtained during repeat imaging sessions on the same or different days (between session reproducibility). This is of particular relevance for group comparisons with healthy volunteers, and longitudinal and interventional studies where DTI can be used as a non-invasive imaging biomarker of disease progression or response to therapy. The rational design and interpretation of such studies is hampered by lack of knowledge regarding how the variability of DTI measurements in data obtained during the same scanning session differs when compared with similar data obtained during a different session or day. In studies where consecutive measurements are performed on each subject under resting and experimental conditions problems associated with variation between subjects due to individual differences (intersubject variability) can be limited. However, baseline DTI measurements may vary within an individual patient (intrasubject variability) and limit the ability to detect significant changes over time or following a therapeutic intervention. Where DTI is repeated after several days or weeks in different imaging sessions the measurements may vary within an individual patient even in the absence of disease progression due to a combination of intrasubject and scanner variability [29], [30]. Without knowledge of such differences it is difficult to accurately determine the clinical significance of pathophysiological changes, as they evolve following various causes of brain injury or disease.

The aim of these studies was to provide reference data on intersubject variability and reproducibility of fractional anisotropy, apparent diffusion coefficient, radial and axial diffusivity measurements in a group of healthy volunteers. These data will inform the design of interventional studies, where repeated measurements are conducted within the same session, and longitudinal studies, where assessments are repeated over time in several different imaging sessions.

## Materials and Methods

### Ethics statement

Ethical approval was obtained from the Cambridgeshire 2 Research Ethics Committee (reference number 97/290), and written informed consent was obtained from all volunteers in accordance with the Declaration of Helsinki.

### Imaging data acquisition

Twenty six healthy volunteers without any history of neuropsychiatric disorder or substance abuse underwent imaging using a 3T Siemens Verio MRI scanner (Siemens AG, Erlangen, Germany) within the Wolfson Brain Imaging Centre (WBIC), University of Cambridge. All volunteers were right handed (ten males and sixteen females) with mean (range) age of 34 (25–44) years, and employed by Cambridge University Hospitals NHS Trust. Each subject was requested to attend two imaging sessions and undergo DTI twice during each session. Twenty-two volunteers attended a second imaging session within a mean (range) of 33 (3–181) days. Structural sequences included 3D T1-weighted magnetization prepared rapid gradient echo (MPRAGE), fluid attenuated inversion recovery (FLAIR), gradient echo and dual spin echo (proton density/T2-weighted). The DTI data were acquired using 63 non-collinear directions, b = 1000 s/mm^2^ with one volume acquired without diffusion weighting (b = 0), echo time (TE) 106 ms, repetition time (TR) 11700 ms, 63 slices, field of view 192 mm×92 mm, 2 mm^3^ isotropic voxels, and an acquisition time of 13∶50 minutes. The two DTI sequences were interspersed within the structural sequences at different intervals within each imaging session in order to allow realistic comparison with clinical studies. In a single subject the second DTI dataset from the baseline imaging session was not successfully completed due to scanner malfunction, while four volunteers failed to attend the second imaging session within six months. Imaging data were checked for patient movement, and data sets degraded by motion artefact were excluded [1].

### Image processing

**Figure 1:**
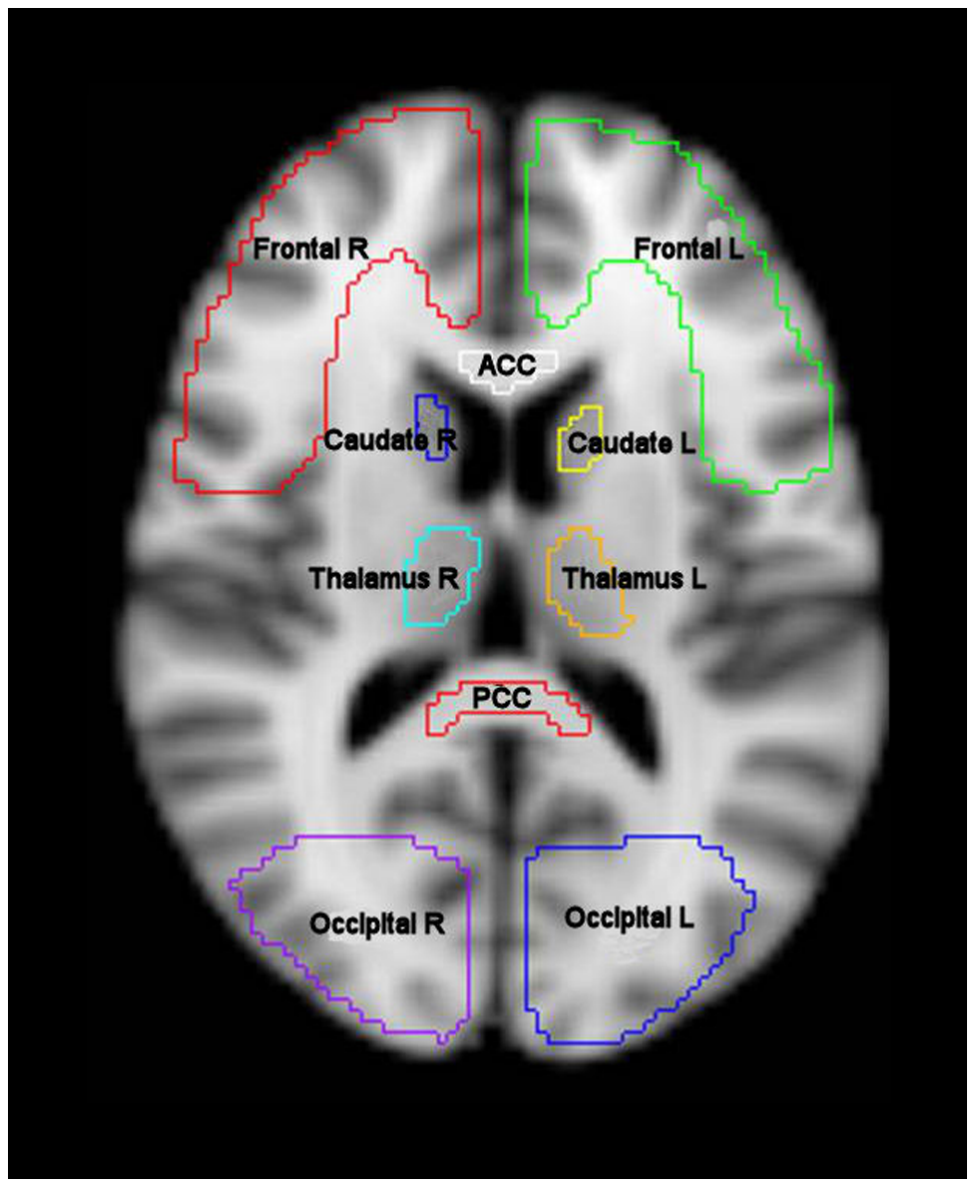
Region of interest template. T1 weighted magnetic resonance image in MNI152 space (2 mm resolution) showing frontal lobe left (Frontal L), frontal lobe right (Frontal R), anterior corpus callosum (ACC), caudate left (Caudate L), caudate right (Caudate R), thalamus left (Thalamus L), thalamus right (Thalamus R), posterior corpus callosum (PCC), occipital left (Occipital L) and occipital right (Occipital R). Additional regions not shown include body corpus callosum, ventral midbrain, dorsal midbrain, forceps minor, forceps major and bilateral regions covering the hippocampus, parietal lobe, temporal lobe, cerebral peduncle, pons, cerebellum, anterior thalamic radiation, superior longitudinal fasciculus, inferior longitudinal fasciculus, cingulum, uncinate fasciculus and corticospinal tract.

Fractional anisotropy (FA), apparent diffusion coefficient (ADC) and axial (AD) maps were created using the Oxford Centre for functional MRI of the brain FSL Diffusion Toolbox [31], [32], while radial (RD) diffusivity values were calculated as the mean of the second and third eigenvalues. To aid coregistration, the skull and extracranial soft tissue were stripped from the T1 weighted image using the Brain Extraction Tool of FSL [33]. The diffusion weighted data were normalized using a two-step approach. First, volunteer T1 weighted images were coregistered to the Montreal Neurological Institute 152 (MNI152) template using the vtkCISG normalized mutual information algorithm [34]. Using the b = 0 image the diffusion weighted data were coregistered to the subjects own T1 weighted image obtained during the same session. The transformation matrix normalizing the MPRAGE was then applied to the diffusion weighted data. Regions of interest (ROIs) from the Harvard Oxford subcortical and MNI structural probabilistic atlases available within FSL were applied in normalised space (figure 1) [35], [36]. All normalised images were inspected using FSL View by a single experienced clinical investigator (TV) to confirm that data processing had completed successfully and that the ROIs were aligned and corresponded to the regions specified. The ROI template was modified by erosion of a single voxel using fslmaths to improve spatial localisation and reduce the impact of coregistration, normalisation and partial volume errors. The FA, mean ADC, AD and RD values for the different ROIs were calculated using in-house software using Matlab (Mathworks, Natick, USA).

### Analysis Strategy

The baseline data from all 26 volunteers were used to calculate the intersubject variability, while within session and between session reproducibility were calculated from available data. The acquisition of two sets of imaging data in each of the two imaging sessions allows the calculation of four independent sets of DTI data, which could be used to assess the reproducibility of measurements. We used the average SD for all DTI measurements obtained in 26 volunteers in both sessions to calculate the population 95% prediction interval (PI) for zero change (using two SD values). These calculated thresholds are prediction intervals for assuming no changes from zero with the repeat DTI measurement rather than confidence intervals for variability of the measurement. Although these average data are extremely useful, the calculated SD could vary within different sessions and particular ROIs within subjects. It would therefore be helpful to have a more specific measure of variability within a session (within session reproducibility), and preferably for each ROI. While this is possible, the small sample numbers (two readings obtained in each of the two sessions) means that a conventional threshold of change greater than 2SD cannot be used to assess the statistical significance of changes in this context. While any estimate of variance based on a *t* distribution with two degrees of freedom must be treated with caution, statistical theory suggests that an estimate of the 95% prediction interval for zero change may be provided by a threshold of 4.3 SDs. These within session measurements could therefore be used to assess the significance of the changes in DTI parameters following a therapeutic intervention within the same imaging session.

### Statistical analysis

Statistical analyses were conducted using Statview (Version 5, 1998, SAS Institute Inc., Cary, North Carolina, USA) and SPSS^®^ Statistics Version 21 (IBM ^®^ Corporation, New York, United States). All data are expressed and displayed as mean and standard deviation (SD), unless otherwise stated. To compare the reproducibility of DTI measurements the SD and coefficient of variation (CoV) (CoV  =  SD/mean) of measurements were calculated within each ROI. Data were compared using paired t-tests, factorial analysis of variance (ANOVA) and intraclass correlation (ICC) as appropriate. Using ANOVA the residual standard deviation was used to calculate the 95% prediction interval for zero change of repeat DTI studies. All *p* values are quoted after Bonferroni corrections for multiple comparisons (where appropriate).

## Results

### Intersubject variability of diffusion tensor imaging metrics

The intersubject variability of DTI measurements is displayed in Tables 1 and 2 for the predominantly white matter and mixed cortical and deep grey matter regions. The intersubject variability was high for all the calculated parameters with a mean (range) CoV across the ROIs for FA of 7.9 (3.3–31.7%) and 6.8 (3.3–19.2%), ADC of 7.3 (2.4–33.7%) and 7.1 (1.8–30.9%), AD of 4.5 (1.5–15.0%) and 6.0 (1.9–27.4%) and RD of 12.4 (3.6–63.2%) and 8.1 (2.6–33.3%) for the white matter and mixed cortical and deep grey matter regions respectively.

**Table 1:** Intersubject variability for diffusion tensor imaging measurements for predominantly white matter regions of interest (ROI).

Region of Interest (ROI)	FA (mm^2^/second)			ADC (mm^2^/second)			Axial diffusivity (mm^2^/second)			Radial diffusivity (mm^2^/second)		
	Mean	SD	CoV (%)	Mean	SD	CoV (%)	Mean	SD	CoV (%)	Mean	SD	CoV (%)
**Ant corpus callosum**	0.667162	0.079716	11.9	0.000828	0.000052	6.2	0.001604	0.000071	4.4	0.000440	0.000092	20.9
**Body corpus callosum**	0.564869	0.179244	31.7	0.000997	0.000187	18.7	0.001678	0.000091	5.4	0.000657	0.000286	43.5
**Post corpus callosum**	0.695110	0.101172	14.6	0.000993	0.000335	33.7	0.001812	0.000271	15.0	0.000584	0.000369	63.2
**Ant thalamic radiation left**	0.407562	0.019832	4.9	0.000833	0.000037	4.5	0.001198	0.000031	2.6	0.000652	0.000042	6.4
**Ant thalamic radiation right**	0.366557	0.019831	5.4	0.000936	0.000046	5.0	0.001274	0.000036	2.9	0.000768	0.000052	6.7
**Sup longitudinal fasciculus left**	0.345421	0.012185	3.5	0.000845	0.000027	3.2	0.001135	0.000027	2.4	0.000699	0.000029	4.1
**Sup longitudinal fasciculus right**	0.371603	0.012607	3.4	0.000828	0.000023	2.7	0.001146	0.000023	2.0	0.000669	0.000024	3.6
**Inf longitudinal fasciculus left**	0.387245	0.017434	4.5	0.000817	0.000020	2.4	0.001171	0.000018	1.5	0.000641	0.000026	4.0
**Inf longitudinal fasciculus right**	0.413091	0.019417	4.7	0.000860	0.000029	3.4	0.001258	0.000033	2.6	0.000660	0.000034	5.2
**Cingulum left**	0.301153	0.035238	11.7	0.000900	0.000051	5.6	0.001184	0.000049	4.1	0.000762	0.000058	7.6
**Cingulum right**	0.300470	0.051387	17.1	0.001013	0.000098	9.7	0.001311	0.000086	6.5	0.000861	0.000113	13.1
**Uncinate fasciculus left**	0.399732	0.018942	4.7	0.000816	0.000021	2.6	0.001177	0.000027	2.3	0.000636	0.000024	3.8
**Uncinate fasciculus right**	0.373047	0.022351	6.0	0.000915	0.000051	5.5	0.001273	0.000046	3.6	0.000736	0.000057	7.7
**Corticospinal tract left**	0.485927	0.016789	3.5	0.000838	0.000036	4.3	0.001275	0.000034	2.7	0.000621	0.000037	6.0
**Corticospinal tract right**	0.485927	0.018213	3.7	0.000819	0.000034	4.2	0.001256	0.000030	2.4	0.000603	0.000035	5.9
**Forceps Minor**	0.391980	0.015603	4.0	0.000895	0.000032	3.6	0.001269	0.000038	3.0	0.000706	0.000035	5.0
**Forceps Major**	0.410602	0.033072	8.1	0.000934	0.000077	8.2	0.001354	0.000082	6.0	0.000724	0.000082	11.3
**Ventral Midbrain**	0.575076	0.065234	11.3	0.000806	0.000102	12.6	0.001405	0.000147	10.4	0.000506	0.000089	17.5
**Dorsal Midbrain**	0.535710	0.037926	7.1	0.000778	0.000044	5.6	0.001262	0.000070	5.5	0.000536	0.000047	8.7
**Cerebral peduncle left**	0.504359	0.023937	4.7	0.000701	0.000027	3.9	0.001136	0.000041	3.6	0.000483	0.000027	5.6
**Cerebral peduncle right**	0.527349	0.017429	3.3	0.000683	0.000023	3.4	0.001132	0.000030	2.6	0.000459	0.000026	5.6
**Pons left**	0.528268	0.030525	5.8	0.000816	0.000091	11.2	0.001308	0.000094	7.2	0.000570	0.000092	16.2
**Pons right**	0.545961	0.027195	5.0	0.000792	0.000065	8.3	0.001300	0.000062	4.8	0.000537	0.000071	13.2
**Mean**	0.460182	0.038056	7.9	0.000854	0.000066	7.3	0.001301	0.000062	4.5	0.000631	0.000076	12.4

Intersubject variability for Fractional anisotropy (FA), apparent diffusion coefficient (ADC), axial and radial diffusivity. Data displayed were obtained in 26 subjects and show mean, standard deviation (SD) and coefficient of variation (CoV).

**Table 2:** Intersubject variability for diffusion tensor imaging measurements for mixed cortical and deep grey matter regions of interest (ROI).

Region of Interest (ROI)	FA (mm^2^/second)			ADC (mm^2^/second)			Axial diffusivity (mm^2^/second)			Radial diffusivity (mm^2^/second)		
	Mean	SD	CoV (%)	Mean	SD	CoV (%)	Mean	SD	CoV (%)	Mean	SD	CoV (%)
**Caudate left**	0.245963	0.047207	19.2	0.001230	0.000379	30.9	0.001473	0.000403	27.4	0.001108	0.000369	33.3
**Caudate right**	0.293710	0.041241	14.0	0.000770	0.000106	13.8	0.000999	0.000116	11.6	0.000655	0.000106	16.2
**Thalamus left**	0.344843	0.016470	4.8	0.000781	0.000023	2.9	0.001054	0.000024	2.3	0.000645	0.000026	4.0
**Thalamus right**	0.352215	0.017537	5.0	0.000756	0.000013	1.8	0.001029	0.000020	1.9	0.000619	0.000016	2.6
**Hippocampus left**	0.284409	0.017636	6.2	0.001030	0.000061	5.9	0.001314	0.000059	4.5	0.000887	0.000063	7.2
**Hippocampus right**	0.291187	0.016893	5.8	0.001113	0.000073	6.6	0.001421	0.000073	5.2	0.000959	0.000075	7.8
**Frontal lobe left**	0.248031	0.010789	4.4	0.001015	0.000046	4.5	0.001234	0.000049	4.0	0.000905	0.000045	5.0
**Frontal lobe right**	0.241779	0.008078	3.3	0.001038	0.000048	4.6	0.001255	0.000049	3.9	0.000930	0.000048	5.2
**Parietal lobe left**	0.261730	0.010499	4.0	0.001016	0.000055	5.4	0.001250	0.000055	4.4	0.000899	0.000055	6.2
**Parietal lobe right**	0.260676	0.009041	3.5	0.001038	0.000051	4.9	0.001271	0.000051	4.0	0.000921	0.000051	5.5
**Occipital lobe left**	0.247951	0.014531	5.9	0.000952	0.000049	5.2	0.001171	0.000051	4.3	0.000842	0.000049	5.8
**Occipital lobe right**	0.240892	0.010980	4.6	0.000996	0.000053	5.3	0.001213	0.000054	4.5	0.000887	0.000052	5.9
**Temporal lobe left**	0.249815	0.017455	7.0	0.000903	0.000040	4.5	0.001127	0.000047	4.2	0.000791	0.000038	4.9
**Temporal lobe right**	0.257036	0.013190	5.1	0.000963	0.000027	2.8	0.001199	0.000026	2.2	0.000845	0.000028	3.4
**Cerebellum left**	0.234565	0.020717	8.8	0.000839	0.000063	7.5	0.001038	0.000069	6.6	0.000739	0.000060	8.2
**Cerebellum right**	0.230964	0.018121	7.8	0.000834	0.000058	7.0	0.001029	0.000057	5.5	0.000737	0.000060	8.1
**Mean**	0.267860	0.018149	6.8	0.000955	0.000072	7.1	0.001192	0.000075	6.0	0.000836	0.000071	8.1

Intersubject variability for Fractional anisotropy (FA), apparent diffusion coefficient (ADC), axial and radial diffusivity. Data displayed were obtained in 26 subjects and show mean, standard deviation (SD) and coefficient of variation (CoV).

### Within session and between session reproducibility of diffusion tensor imaging

**Figure 2:**
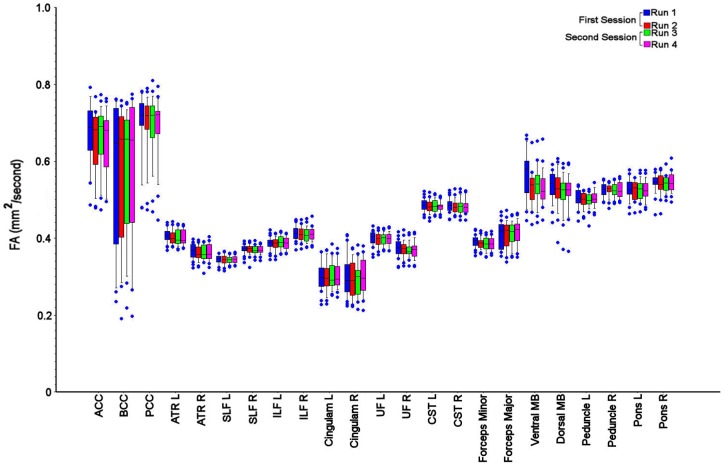
Variability in fractional anisotropy measurements. Box and whisker plot for fractional anisotropy values (mm^2^/second) for the white matter region of interest (ROI) measurements. The spread of data within each ROI reflects inter subject variation, while the difference between runs 1–2 and 3–4 reflects within session reproducibility, and the change from first to second sessions reflects between session reproducibility. The central lines in each box denote median values, the lower and upper boundaries the 25th and 75th centile, the error bars the 10th and 90th centile, and the closed circles outlying data points. Anterior corpus callosum (ACC), body corpus callosum (BCC), posterior corpus callosum (PCC), left anterior thalamic radiation (ATR L), right anterior thalamic radiation (ATR R), left superior longitudinal fasciculus (SLF L), right superior longitudinal fasciculus (SLF R), left inferior longitudinal fasciculus (ILF L), right inferior longitudinal fasciculus (ILF R), left cingulum (Cingulum L), right cingulum (Cingulum R), left uncinate fasciculus (UFL), right uncinate fasciculus (UFR), left corticospinal tract (CST L), right corticospinal tract (CST R), dorsal midbrain (dorsal MB), ventral midbrain (ventral midbrain), left cerebral peduncle (CP L), right cerebral peduncle (CP R), left pons (pons L) and right pons (pons R).

The ROI data for within and between session reproducibility were variable across the different brain regions, but lower than the values for intersubject variability (Tables 3–6). The within session reproducibility measurements were significantly lower than between session reproducibility measurements for all the DTI parameters (p<0.001, paired ‘t’ test with Bonferroni correction). As an example, the difference between intersubject variability, within and between session reproducibility is displayed for FA in figure 2.

**Table 3:** Within and between session variability of diffusion tensor imaging region of interest measurements.

	FA		ADC		AD		RD	
	Within session reproducibility	Between session reproducibility	Within session reproducibility	Between session reproducibility	Within session reproducibility	Between session reproducibility	Within session reproducibility	Between session reproducibility
**Ant corpus callosum**	2.0×10^−2^±1.6×10^−2^	1.9×10^−2^±1.7×10^−2^	1.2×10^−5^±1.1×10^−5^	1.5×10^−5^±1.2×10^−5^	2.2×10^−5^±2.0×10^−5^	2.5×10^−5^±2.1×10^−5^	1.9×10^−5^±1.9×10^−5^	2.3×10^−5^±2.3×10^−5^
**Body corpus callosum**	1.7×10^−2^±1.5×10^−2^	1.8×10^−2^±1.8×10^−2^	2.3×10^−5^±2.2×10^−5^	3.2×10^−5^±2.8×10^−5^	2.6×10^−5^±2.8×10^−5^	3.5×10^−5^±2.5×10^−5^	2.4×10^−5^±2.4×10^−5^	3.3×10^−5^±3.5×10^−5^
**Post corpus callosum**	1.0×10^−2^±1.5×10^−2^	1.0×10^−2^±8.2×10^−3^	2.2×10^−5^±3.7×10^−5^	2.3×10^−5^±2.4×10^−5^	2.1×10^−5^±2.8×10^−5^	2.3×10^−5^±2.0×10^−5^	2.2×10^−5^±4.1×10^−5^	2.2×10^−5^±2.9×10^−5^
**Ant thalamic radiation left**	3.6×10^−3^ ±5.1×10^−3^	4.6×10^−3^±4.6×10^−3^	6.4×10^−6^±1.0×10^−5^	9.7×10^−6^±9.9×10^−6^	7.4×10^−6^±8.1×10^−6^	1.1×10^−5^±9.2×10^−6^	6.5×10^−6^±1.2×10^−5^	9.6×10^−6^±1.1×10^−5^
**Ant thalamic radiation right**	4.5×10^−3^±6.0×10^−3^	5.3×10^−3^±4.9×10^−3^	8.7×10^−6^±1.7×10^−5^	1.1×10^−5^±1.4×10^−5^	8.8×10^−6^±1.5×10^−5^	1.3×10^−5^±1.2×10^−5^	9.5×10^−6^±1.9×10^−5^	1.2×10^−5^±1.5×10^−5^
**Sup longitudinal fasciculus left**	4.9×10^−3^±1.9×10^−2^	4.4×10^−3^±1.4×10^−2^	5.6×10^−6^±2.2×10^−5^	8.2×10^−6^±1.7×10^−5^	9.1×10^−6^±3.8×10^−5^	1.2×10^−5^±3.0×10^−5^	4.2×10^−6^±1.4×10^−5^	6.7×10^−6^±1.2×10^−5^
**Sup longitudinal fasciculus right**	5.4×10^−3^±1.9×10^−2^	5.9×10^−3^±1.4×10^−2^	3.3×10^−6^±1.0×10^−5^	7.7×10^−6^±1.0×10^−5^	6.6×10^−6^±2.6×10^−5^	1.3×10^−5^±2.2×10^−5^	2.5×10^−6^±3.2×10^−6^	6.0×10^−6^±5.6×10^−6^
**Inf longitudinal fasciculus left**	5.1×10^−3^±1.7×10^−2^	4.9×10^−3^±1.2×10^−2^	3.1×10^−6^±2.7×10^−6^	6.1×10^−6^±5.3×10^−6^	5.7×10^−6^±1.6×10^−5^	7.9×10^−6^±1.4×10^−5^	4.7×10^−6^±9.9×10^−6^	7.3×10^−6^±8.0×10^−6^
**Inf longitudinal fasciculus right**	5.2×10^−3^±1.3×10^−2^	5.1×10^−3^±9.6×10^−3^	4.3×10^−6^±5.5×10^−6^	7.9×10^−6^±6.4×10^−6^	7.0×10^−6^±1.6×10^−5^	9.5×10^−6^±1.3×10^−5^	6.2×10^−6^±1.0×10^−5^	8.9×10^−6^±8.1×10^−6^
**Cingulum left**	7.3×10^−3^±8.3×10^−3^	7.9×10^−3^±7.1×10^−3^	1.2×10^−5^±1.4×10^−5^	1.3×10^−5^±1.0×10^−5^	1.6×10^−5^±2.0×10^−5^	1.6×10^−5^±1.6×10^−5^	1.3×10^−5^±1.5×10^−5^	1.4×10^−5^±1.2×10^−5^
**Cingulum right**	8.0×10^−3^±9.5×10^−3^	9.6×10^−3^±8.3×10^−3^	1.9×10^−5^±1.5×10^−5^	2.2×10^−5^±1.8×10^−5^	2.4×10^−5^±1.8×10^−5^	2.5×10^−5^±1.9×10^−5^	1.9×10^−5^±1.7×10^−5^	2.0×10^−5^±1.8×10^−5^
**Uncinate fasciculus left**	4.6×10^−3^±8.0×10^−3^	5.4×10^−3^±6.7×10^−3^	4.7×10^−6^±5.3×10^−6^	7.3×10^−6^±6.8×10^−6^	6.4×10^−6^±4.9×10^−6^	9.6×10^−6^±8.3×10^−6^	6.6×10^−6^±1.2×10^−5^	9.1×10^−6^±1.0×10^−5^
**Uncinate fasciculus right**	4.8×10^−3^±4.2×10^−3^	4.7×10^−3^±3.4×10^−3^	7.7×10^−6^±9.0×10^−6^	9.0×10^−6^±8.5×10^−6^	8.2×10^−6^±9.4×10^−6^	1.0×10^−5^±8.5×10^−6^	9.7×10^−6^±9.7×10^−6^	1.0×10^−5^±8.9×10^−6^
**Corticospinal tract left**	6.7×10^−3^±2.0×10^−2^	6.5×10^−3^±1.5×10^−2^	6.4×10^−6^±1.0×10^−5^	9.9×10^−6^±1.0×10^−5^	1.0×10^−5^±2.8×10^−5^	1.4×10^−5^±2.2×10^−5^	6.8×10^−6^±1.2×10^−5^	1.0×10^−5^±1.1×10^−5^
**Corticospinal tract right**	6.4×10^−3^±1.5×10^−2^	7.0×10^−3^±1.2×10^−2^	5.3×10^−6^±7.7×10^−6^	9.2×10^−6^±9.6×10^−6^	8.9×10^−6^±2.0×10^−5^	1.4×10^−5^±1.8×10^−5^	6.0×10^−6^±1.3×10^−5^	9.6×10^−6^±1.3×10^−5^
**Forceps Minor**	5.5×10^−3^±6.9×10^−3^	6.9×10^−3^±6.2×10^−3^	7.9×10^−6^±8.4×10^−6^	1.0×10^−5^±8.2×10^−6^	9.4×10^−6^±9.0×10^−6^	1.4×10^−5^±1.1×10^−5^	8.9×10^−6^±1.1×10^−5^	1.2×10^−5^±8.7×10^−6^
**Forceps Major**	5.4×10^−3^±1.5×10^−2^	4.3×10^−3^±1.1×10^−2^	4.6×10^−6^±6.6×10^−6^	7.3×10^−6^±6.7×10^−6^	7.9×10^−6^±2.1×10^−5^	1.0×10^−5^±1.7×10^−5^	5.6×10^−6^±7.5×10^−6^	7.4×10^−6^±5.8×10^−6^
**Ventral Midbrain**	3.0×10^−2^±3.7×10^−2^	2.6×10^−2^±2.5×10^−2^	3.0×10^−5^±2.6×10^−5^	4.3×10^−5^±3.4×10^−5^	5.1×10^−5^±5.1×10^−5^	6.3×10^−5^±4.4×10^−5^	2.2×10^−5^±1.8×10^−5^	2.9×10^−5^±2.3×10^−5^
**Dorsal Midbrain**	1.3×10^−2^±1.1×10^−2^	2.1×10^−2^±1.8×10^−2^	1.3×10^−5^±1.4×10^−5^	3.1×10^−5^±3.7×10^−5^	1.6×10^−5^±1.6×10^−5^	3.5×10^−5^±3.5×10^−5^	1.9×10^−5^±1.9×10^−5^	2.5×10^−5^±2.8×10^−5^
**Cerebral peduncle left**	7.5×10^−3^±7.6×10^−3^	9.3×10^−3^±7.4×10^−3^	8.8×10^−6^±1.1×10^−5^	1.5×10^−5^±1.3×10^−5^	1.4×10^−5^±1.5×10^−5^	2.0×10^−5^±1.5×10^−5^	9.4×10^−6^±1.0×10^−5^	1.4×10^−5^±1.3×10^−5^
**Cerebral peduncle right**	5.9×10^−3^±4.3×10^−3^	6.9×10^−3^±5.0×10^−3^	7.4×10^−6^±6.2×10^−6^	1.1×10^−5^±8.7×10^−6^	1.2×10^−5^±1.0×10^−5^	1.7×10^−5^±1.2×10^−5^	8.5×10^−6^±8.3×10^−6^	1.3×10^−5^±1.6×10^−5^
**Pons left**	8.9×10^−3^±8.1×10^−3^	1.2×10^−2^±1.1×10^−2^	1.3×10^−5^±1.2×10^−5^	3.1×10^−5^±2.2×10^−5^	1.6×10^−5^±1.5×10^−5^	3.6×10^−5^±2.3×10^−5^	1.7×10^−5^±1.6×10^−5^	2.8×10^−5^±3.0×10^−5^
**Pons right**	7.7×10^−3^±7.3×10^−3^	1.3×10^−2^±7.4×10^−3^	1.1×10^−5^±9.3×10^−6^	2.7×10^−5^±2.1×10^−5^	1.5×10^−5^±1.3×10^−5^	2.7×10^−5^±2.4×10^−5^	1.4×10^−5^±1.2×10^−5^	2.7×10^−5^±2.6×10^−5^
**Mean**	7.1×10^−3^±1.3×10^−2^	8.3×10^−3^±1.1×10^−2^	1.0×10^−5^±1.6×10^−5^	1.7×10^−5^±2.1×10^−5^	1.4×10^−5^±2.2×10^−5^	2.1×10^−5^±2.5×10^−5^	1.1×10^−5^±1.7×10^−5^	1.6×10^−5^±2.1×10^−5^

Individual white matter region of interest measurements for within session reproducibility obtained in the first and second imaging sessions in 26 and 22 subjects respectively, and the between session reproducibility for those 22 subjects who underwent imaging at both sessions. Data displayed are standard deviation of measurements for fractional anisotropy (FA), apparent diffusion coefficient (ADC), axial (AD) and radial (RD) diffusivity.

**Table 4:** Within and between session variability of diffusion tensor imaging region of interest measurements.

	FA	ADC				AD		RD	
	Within session reproducibility	Between session reproducibility	Within session reproducibility	Between session reproducibility	Within session reproducibility		Between session reproducibility	Within session reproducibility	Between session reproducibility
**Caudate left**	9.0×10^−3^±7.5×10^−3^	9.1×10^−3^±8.5×10^−3^	4.1×10^−5^±4.1×10^−5^	4.2×10^−5^±3.5×10^−5^	4.8×10^−5^±4.5×10^−5^		4.9×10^−5^±4.0×10^−5^	3.1×10^−5^±3.5×10^−5^	3.0×10^−5^±2.7×10^−5^
**Caudate right**	9.2×10^−3^±8.1×10^−3^	9.7×10^−3^±7.2×10^−3^	1.4×10^−5^±1.9×10^−5^	1.9×10^−5^±3.3×10^−5^	1.5×10^−5^±1.9×10^−5^		2.1×10^−5^±3.5×10^−5^	1.3×10^−5^±1.9×10^−5^	1.7×10^−5^±3.2×10^−5^
**Thalamus left**	6.1×10^−3^±4.5×10^−3^	6.8×10^−3^±4.9×10^−3^	7.9×10^−6^±6.8×10^−6^	1.1×10^−5^±8.4×10^−6^	1.1×10^−5^±8.3×10^−6^		1.6×10^−5^±1.1×10^−5^	7.8×10^−6^±8.2×10^−6^	1.1×10^−5^±1.6×10^−5^
**Thalamus right**	5.7×10^−3^±4.9×10^−3^	6.9×10^−3^±5.7×10^−3^	6.2×10^−6^±4.8×10^−6^	8.8×10^−6^±6.1×10^−6^	8.2×10^−6^±7.8×10^−6^		1.4×10^−5^±9.8×10^−6^	7.5×10^−6^±7.9×10^−6^	1.1×10^−5^±1.7×10^−5^
**Hippocampus left**	4.2×10^−3^±4.8×10^−3^	4.8×10^−3^±4.3×10^−3^	7.0×10^−6^±8.4×10^−6^	1.3×10^−5^±1.1×10^−5^	8.5×10^−6^±8.1×10^−6^		1.4×10^−5^±1.2×10^−5^	8.2×10^−6^±1.2×10^−5^	1.4×10^−5^±2.0×10^−5^
**Hippocampus right**	4.7×10^−3^±6.7×10^−3^	5.7×10^−3^±3.8×10^−3^	9.7×10^−6^±9.8×10^−6^	1.6×10^−5^±1.2×10^−5^	1.1×10^−5^±9.0×10^−6^		1.6×10^−5^±1.3×10^−5^	9.2×10^−6^±1.2×10^−5^	1.6×10^−5^±1.9×10^−5^
**Frontal lobe left**	3.1×10^−3^±3.7×10^−3^	4.1×10^−3^±4.1×10^−3^	9.1×10^−6^±9.6×10^−6^	2.0×10^−5^±1.6×10^−5^	1.1×10^−5^±1.3×10^−5^		2.3×10^−5^±2.0×10^−5^	9.6×10^−6^±8.0×10^−6^	1.5×10^−5^±1.3×10^−5^
**Frontal lobe right**	3.2×10^−3^±3.0×10^−3^	3.9×10^−3^±3.6×10^−3^	9.0×10^−6^±8.4×10^−6^	2.0×10^−5^±1.7×10^−5^	1.1×10^−5^±1.1×10^−5^		2.4×10^−5^±2.1×10^−5^	8.9×10^−6^±7.7×10^−6^	1.6×10^−5^±1.4×10^−5^
**Parietal lobe left**	3.7×10^−3^±4.3×10^−3^	4.9×10^−3^±4.9×10^−3^	7.3×10^−6^±8.4×10^−6^	1.3×10^−5^±1.2×10^−5^	9.1×10^−6^±1.1×10^−5^		1.7×10^−5^±1.6×10^−5^	7.1×10^−6^±8.2×10^−6^	1.3×10^−5^±1.3×10^−5^
**Parietal lobe right**	2.4×10^−3^±2.8×10^−3^	4.0×10^−3^±3.6×10^−3^	4.5×10^−6^±3.3×10^−6^	1.2×10^−5^±1.2×10^−5^	5.1×10^−6^±4.3×10^−6^		1.4×10^−5^±1.4×10^−5^	5.7×10^−6^±6.3×10^−6^	1.2×10^−5^±1.4×10^−5^
**Occipital lobe left**	5.0×10^−3^±6.8×10^−3^	5.5×10^−3^±5.2×10^−3^	6.5×10^−6^±6.8×10^−6^	1.3×10^−5^±9.4×10^−6^	9.3×10^−6^±1.0×10^−5^		1.5×10^−5^±1.2×10^−5^	6.4×10^−6^±7.2×10^−6^	1.2×10^−5^±1.4×10^−5^
**Occipital lobe right**	3.7×10^−3^±4.7×10^−3^	4.4×10^−3^±4.8×10^−3^	6.3×10^−6^±6.6×10^−5^	1.3×10^−5^±1.2×10^−5^	8.8×10^−6^±9.2×10^−6^		1.5×10^−5^±1.5×10^−5^	7.4×10^−6^±8.1×10^−6^	1.4×10^−5^±1.7×10^−5^
**Temporal lobe left**	6.7×10^−3^±8.5×10^−3^	6.7×10^−3^±7.5×10^−3^	1.0×10^−5^±9.4×10^−6^	1.3×10^−5^±1.1×10^−5^	1.4×10^−5^±1.4×10^−5^		1.7×10^−5^±1.6×10^−5^	8.6×10^−6^±8.2×10^−6^	1.4×10^−5^±1.6×10^−5^
**Temporal lobe right**	4.4×10^−3^±5.1×10^−3^	4.7×10^−3^±4.8×10^−3^	7.0×10^−6^±5.4×10^−6^	1.1×10^−5^±8.5×10^−6^	8.9×10^−6^±8.1×10^−6^		1.3×10^−5^±1.0×10^−5^	1.3×10^−5^±4.4×10^−5^	1.5×10^−5^±3.6×10^−5^
**Cerebellum left**	4.5×10^−3^±4.7×10^−3^	1.3×10^−2^±1.3×10^−2^	1.2×10^−5^±1.5×10^−5^	3.5×10^−5^±3.8×10^−5^	1.5×10^−5^±1.9×10^−5^		4.4×10^−5^±4.9×10^−5^	1.3×10^−5^±1.4×10^−5^	2.4×10^−5^±2.7×10^−5^
**Cerebellum right**	4.0×10^−3^±4.4×10^−3^	1.2×10^−2^±1.2×10^−2^	8.9×10^−6^±1.1×10^−5^	3.1×10^−5^±3.4×10^−5^	1.2×10^−5^±1.3×10^−5^		4.0×10^−5^±4.2×10^−5^	1.2×10^−5^±1.2×10^−5^	2.4×10^−5^±2.4×10^−5^
**Mean**	5.0×10^−3^±5.8×10^−3^	6.6×10^−3^±7.2×10^−3^	1.0×10^−5^±1.6×10^−5^	1.8×10^−5^±2.2×10^−5^	1.3×10^−5^±1.8×10^−5^		2.2×10^−5^±2.7×10^−5^	1.1×10^−5^±1.8×10^−5^	1.6×10^−5^±2.2×10^−5^

Individual mixed cortical and deep grey matter region of interest measurements for within session reproducibility obtained in the first and second imaging sessions in 26 and 22 subjects respectively, and the between session reproducibility for those 22 subjects who underwent imaging at both sessions. Data displayed are standard deviation of measurements for fractional anisotropy (FA), apparent diffusion coefficient (ADC), axial (AD) and radial (RD) diffusivity.

**Table 5:** Within and between session variability of diffusion tensor imaging region of interest measurements.

	FA		ADC		AD		RD	
	Within session reproducibility	Between session reproducibility	Within session reproducibility	Between session reproducibility	Within session reproducibility	Between session reproducibility	Within session reproducibility	Between session reproducibility
**Ant corpus callosum**	3.2±2.6	3.0±2.7	1.5±1.3	1.9±1.4	1.4±1.3	1.6±1.3	4.3±4.6	5.2±5.0
**Body corpus callosum**	3.6±3.6	3.8±4.2	2.4±2.4	3.1±2.7	1.6±1.7	2.1±1.6	3.9±4.6	5.2±5.7
**Post corpus callosum**	1.6±2.1	1.5±1.3	2.2±3.4	2.2±2.1	1.1±1.4	1.3±1.0	3.8±6.6	4.0±4.2
**Ant thalamic radiation left**	0.9±1.3	1.1±1.1	0.8±1.2	1.2±1.2	0.6±0.7	0.9±0.8	1.0±1.8	1.4±1.6
**Ant thalamic radiation right**	1.3±1.6	1.5±1.4	0.9±1.8	1.2±1.4	0.7±1.2	1.0±0.9	1.2±2.3	1.5±1.8
**Sup longitudinal fasciculus left**	1.7±7.4	1.4±5.5	0.7±3.0	1.0±2.3	0.9±4.0	1.1±3.1	0.6±2.3	1.0±1.8
**Sup longitudinal fasciculus right**	1.7±6.3	1.7±4.7	0.4±1.4	1.0±1.3	0.6±2.6	1.1±2.2	0.4±0.5	0.9±0.9
**Inf longitudinal fasciculus left**	1.4±5.0	1.3±3.6	0.4±0.3	0.7±0.7	0.5±1.4	0.7±1.2	0.7±1.5	1.1±1.2
**Inf longitudinal fasciculus right**	1.3±3.5	1.3±2.5	0.5±0.7	0.9±0.7	0.6±1.3	0.8±1.1	0.9±1.6	1.4±1.2
**Cingulum left**	2.5±2.8	2.7±2.3	1.3±1.5	1.4±1.1	1.3±1.6	1.4±1.3	1.7±1.9	1.9±1.6
**Cingulum right**	2.7±3.0	3.2±2.6	1.9±1.5	2.2±1.8	1.8±1.4	1.9±1.5	2.2±1.9	2.4±2.0
**Uncinate fasciculus left**	1.2±2.1	1.4±1.8	0.6±0.6	0.9±0.8	0.5±0.4	0.8±0.7	1.0±1.8	1.4±1.5
**Uncinate fasciculus right**	1.3±1.2	1.3±0.9	0.8±1.0	1.0±0.9	0.6±0.8	0.8±0.7	1.3±1.4	1.4±1.2
**Corticospinal tract left**	1.5±4.8	1.4±3.5	0.8±1.4	1.2±1.3	0.8±2.4	1.1±1.9	1.1±1.9	1.9±1.8
**Corticospinal tract right**	1.4±3.6	1.5±2.9	0.7±1.0	1.1±1.2	0.7±1.7	1.1±1.5	1.0±2.1	1.6±2.1
**Forceps Minor**	1.5±1.9	1.8±1.7	0.9±0.9	1.1±0.9	0.7±0.7	1.1±0.8	1.3±1.5	1.6±1.2
**Forceps Major**	1.5±4.4	1.1±3.2	0.5±0.8	0.8±0.8	0.6±1.8	0.8±1.4	0.8±1.1	1.0±0.8
**Ventral Midbrain**	5.5±6.6	4.7±4.4	3.9±3.5	5.7±4.5	3.8±4.0	4.7±3.4	4.3±3.6	5.8±4.5
**Dorsal Midbrain**	2.5±2.2	4.0±3.4	1.7±1.7	3.8±4.4	1.3±1.2	2.7±2.5	3.3±3.0	4.5±4.9
**Cerebral peduncle left**	1.5±1.6	1.8±1.5	1.3±1.6	2.2±1.9	1.2±1.4	1.8±1.4	1.9±2.1	3.0±2.6
**Cerebral peduncle right**	1.1±0.9	1.3±0.9	1.1±0.9	1.7±1.3	1.0±0.9	1.5±1.1	1.8±1.7	2.8±3.3
**Pons left**	1.7±1.6	2.3±2.1	1.5±1.5	3.8±2.8	1.2±1.2	2.8±1.8	2.9±2.6	4.9±5.4
**Pons right**	1.4±1.4	2.3±1.4	1.4±1.2	3.4±2.6	1.2±1.0	2.1±1.8	2.6±2.0	4.8±4.4
**Mean**	1.9±3.7	2.1±3.0	1.2±1.9	1.9±2.4	1.1±1.9	1.5±1.9	1.9±2.9	2.6±3.5

Individual white matter region of interest measurements for within session reproducibility obtained in the first and second imaging sessions in 26 and 22 subjects respectively, and the between session reproducibility for those 22 subjects who underwent imaging at both sessions. Data displayed are percentage coefficient of variation of measurements for fractional anisotropy (FA), apparent diffusion coefficient (ADC), axial (AD) and radial (RD) diffusivity.

**Table 6:** Within and between session variability of diffusion tensor imaging region of interest measurements.

	FA		ADC		AD		RD	
	Within session reproducibility	Between session reproducibility	Within session reproducibility	Between session reproducibility	Within session reproducibility	Between session reproducibility	Within session reproducibility	Between session reproducibility
**Caudate left**	3.8±3.4	3.9±4.0	3.3±3.3	3.6±3.0	3.2±2.9	3.4±2.8	2.9±3.2	3.0±2.8
**Caudate right**	3.2±2.9	3.4±2.5	1.7±2.2	2.2±3.0	1.4±1.7	2.0±2.6	1.9±2.5	2.2±3.4
**Thalamus left**	1.8±1.3	2.0±1.5	1.0±0.9	1.4±1.1	1.0±0.8	1.5±1.0	1.2±1.2	1.6±2.3
**Thalamus right**	1.6±1.4	2.0±1.6	0.8±0.6	1.2±0.8	0.8±0.8	1.3±1.0	1.2±1.1	1.8±2.5
**Hippocampus left**	1.5±1.7	1.7±1.6	0.7±0.8	1.2±1.1	0.6±0.6	1.1±0.9	0.9±1.3	1.6±2.2
**Hippocampus right**	1.6±2.3	2.0±1.4	0.9±0.9	1.4±1.1	0.8±0.6	1.1±0.9	1.0±1.3	1.7±2.0
**Frontal lobe left**	1.2±1.5	1.7±1.6	0.9±1.0	1.9±1.6	0.9±1.0	1.9±1.6	1.1±0.9	1.6±1.5
**Frontal lobe right**	1.4±1.2	1.6±1.5	0.9±0.8	2.0±1.7	0.9±0.9	1.9±1.7	1.0±0.8	1.7±1.5
**Parietal lobe left**	1.4±1.6	1.9±1.9	0.7±0.8	1.3±1.2	0.7±0.9	1.3±1.2	0.8±0.9	1.4±1.4
**Parietal lobe right**	0.9±1.1	1.6±1.4	0.4±0.3	1.2±1.2	0.4±0.3	1.2±1.2	0.6±0.6	1.3±1.5
**Occipital lobe left**	2.1±2.8	2.2±2.1	0.7±0.7	1.3±1.0	0.8±0.8	1.3±1.0	0.7±0.8	1.4±1.6
**Occipital lobe right**	1.6±2.0	1.9±2.0	0.6±0.6	1.3±1.2	0.7±0.7	1.3±1.2	0.8±0.8	1.5±1.8
**Temporal lobe left**	2.7±3.4	2.8±3.0	1.1±1.0	1.5±1.2	1.3±1.2	1.5±1.4	1.1±1.0	1.8±1.9
**Temporal lobe right**	1.7±2.0	1.9±1.9	0.7±0.6	1.2±0.9	0.7±0.7	1.1±0.9	1.8±6.9	1.9±5.3
**Cerebellum left**	2.1±2.5	5.9±6.4	1.5±2.1	4.3±4.9	1.5±2.1	4.4±5.1	1.7±1.9	3.3±3.8
**Cerebellum right**	1.9±2.2	5.5±6.3	1.1±1.4	3.8±4.0	1.2±1.4	4.0±4.2	1.6±1.7	3.2±3.1
**Mean**	1.9±2.3	2.6±3.3	1.1±1.5	1.9±2.4	1.1±1.4	1.9±2.4	1.3±2.3	1.9±2.7

Individual mixed cortical and deep grey region of interest measurements for within session reproducibility obtained in the first and second imaging sessions in 26 and 22 subjects respectively, and the between session reproducibility for those 22 subjects who underwent imaging at both sessions. Data displayed are percentage coefficient of variation of measurements for fractional anisotropy (FA), apparent diffusion coefficient (ADC), axial (AD) and radial (RD) diffusivity.

The mean (range) ROI ICC for within session measurements were for FA 0.79 (0.46–0.99) and 0.81 (0.57–0.93), ADC 0.91 (0.73–0.99) and 0.92 (0.74–0.98), AD 0.82 (0.59–0.98) and 0.89 (0.68–0.98), and for RD 0.89 (0.76–0.99) and 0.91 (0.59–0.99) for the white matter and mixed cortical and deep grey matter regions respectively. The between session measurements were for FA 0.78 (0.56–0.98) and 0.69 (0.42–0.93), ADC 0.79 (0.17–0.99) and 0.78 (0.40–0.98), AD 0.74 (0.47–0.98) and 0.68 (0.19–0.98), and for RD 0.82 (0.46–0.99) and 0.79 (0.48–0.99) for the white matter and mixed cortical and deep grey matter regions respectively.

### Calculation of 95% prediction interval for zero change

Using the four DTI measurements obtained from both sessions we used ANOVA to determine the significance of the differences (Table 7). These confirm that there is a significant difference between regions and subjects, and that there is a significant interaction between brain region and subject. The residual variance of the DTI measurements which could not be accounted for by the known independent variables is shown in Table 7, and the calculated SD for FA, ADC, AD and RD were 1.2×10^−2^, 3.2×10^−5^, 3.2×10^−5^ and 8.4×10^−5^ mm^2^/second respectively. The overall population 95% prediction intervals for zero change (based on two SD values) were therefore 2.4×10^−2^, 6.3×10^−5^, 6.3×10^−5^ and 1.7×10^−4^ mm^2^/second for FA, ADC, AD and RD respectively. The calculated SD for the within session measurements were 7.1×10^−3^, 1.0×10^−5^, 1.4×10^−5^ and 1.1×10^−5^ mm^2^/second for FA, ADC, AD and RD respectively. An estimate of the overall 95% prediction interval for zero change (based on 4.3 SD values) within a single imaging session was therefore 3.1×10^−2^, 4.5×10^−5^, 5.9×10^−5^ and 4.7×10^−5^ mm^2^/second for FA, ADC, AD and RD respectively.

**Table 7:** Analysis of variance table for diffusion tensor imaging parameters.

Parameter	Session	DF	Sum of Squares	Mean Square	F Value	p Value
**FA**	ROI	38	5.4×10^1^	1.4	9.6×10^3^	<.0001
	subject	21	3.5×10^−1^	1.7×10^−2^	1.1×10^2^	<.0001
	ROI * subject	798	5.3	6.6×10^−3^	4.4×10^1^	<.0001
	Residual	2574	3.8×10^−1^	1.5×10^−4^		
**ADC**	ROI	38	4.6×10^−5^	1.2×10^−6^	2.1×10^3^	<.0001
	subject	21	2.2×10^−6^	1.1×10^−7^	1.9×10^2^	<.0001
	ROI * subject	798	2.9×10^−5^	3.6×10^−8^	6.4×10^1^	<.0001
	Residual	2574	1.5×10^−6^	5.7×10^−10^		
**AD**	ROI	38	9.5×10^−5^	2.5×10^−6^	3.0×10^3^	<.0001
	subject	21	1.9×10^−6^	8.9×10^−8^	1.1×10^2^	<.0001
	ROI * subject	798	2.7×10^−5^	3.4×10^−8^	4.1×10^1^	<.0001
	Residual	2574	2.1×10^−6^	8.3×10^−10^		
**RD**	ROI	38	7.3×10^−5^	1.9×10^−6^	2.8×10^2^	<.0001
	subject	21	2.0×10^−6^	9.5×10^−8^	1.4	<.0001
	ROI * subject	798	2.5×10^−5^	3.2×10^−8^	4.6	<.0001
	Residual	2574	1.8×10^−5^	6.9×10^−9^		

Data (mm^2^/second) were obtained from 26 volunteers using the region of interest (ROI) template for fractional anisotropy (FA), apparent diffusion coefficient (ADC), axial diffusivity (AD), and radial diffusivity (RD). Degrees of freedom (DF).

## Discussion

This study provides additional reference data concerning intersubject variability and reproducibility of DTI conducted within the same imaging session (within session) and different imaging sessions (between session) in a group of healthy volunteers. As reported previously [23], we found that intersubject variability was high, with substantial variability across the brain for all the calculated parameters. While the DTI measurements were stable with CoV values of ∼5%, the repeated DTI sequences obtained during the same session (within session) had lower CoV values than those obtained from measurements obtained in a different imaging session separated by up to six months. The calculated 95% prediction intervals for zero change of repeat DTI measurements were similar for the data obtained within the same session and that calculated from all the measurements obtained over both imaging sessions. These prediction intervals can be calculated for individual ROIs and utilised in interventional studies to quantify change within a single imaging session, or to assess the significance of change in longitudinal studies of brain injury and disease.

The factors affecting the reproducibility of DTI parameters include changes within the MR scanner or individual subjects. Features related to the scanner include B_0_ field inhomogeneities, scanner drift, gradient coil stability, signal to noise ratio and software upgrades. Such factors may be more significant when imaging is acquired within different imaging sessions, rather than repeat acquisitions within the same session where such parameters are more likely to be similar. Regular servicing and daily quality assurance measurements seek to ensure that an MR scanner is operating normally. It is obviously necessary to monitor such changes, and where possible, take steps to limit their impact. There were no upgrades or changes in MR scanner hardware or software during the period of this study. In addition, daily signal to noise ratio measurements were not significantly different for the six month period of this study (p = 0.08, Friedman test. Data not shown). While scanner variability is important there are individual subject factors that can induce substantial variability in DTI parameters. These include head movements, and positioning within the scanner field of view. We undertook standard procedures to limit such variability. All subjects were positioned within the head coil according to standard operating procedure and their alignment was confirmed prior to commencing imaging. Following standard imaging for localisation we monitored subject movement, and all data were checked during acquisition and processing for evidence of motion artefact. While no subject was excluded during acquisition or processing in these analyses DTI had to be repeated in one subject during an imaging session due to subject movement. In addition, we performed all analyses following image coregistration and spatial normalisation to MNI standard space. We used a standard ROI template covering the whole brain from the Harvard Oxford subcortical and MNI structural probabilistic atlases available within FSL. While the use of this analysis strategy sought to reduce variability within our comparisons, we eroded the ROI template by a single voxel within FSL in order to improve spatial localisation and reduce the impact of coregistration, normalisation and partial volume errors. Finally, all ROIs were manually inspected to ensure that they were aligned with the imaging data and corresponded to the regions specified. In summary, we considered possible sources of DTI variability within our centre and attempted to limit their impact and ensure that the data we acquired were comparable within and between the different imaging sessions.

While our results for DTI reproducibility are in line with published data [12], [19], [37], we report data specifically concerning the difference between intersubject variability, within session and between session reproducibility. It is useful to consider the sources of variability in DTI data in the setting where we are trying to address the significance of changes between normal physiology and disease states, or changes that are the consequence of a therapeutic intervention. In the first case, the relevant sources of error are the intersubject variability in the patient and volunteer groups. Our data for healthy volunteers are broadly concordant with results from other groups [23], and show that these are high, with mean (range) CoV of 7% (3–32) for FA, 7% (2–34) for ADC, 5% (2–27) for axial diffusivity and 11% (3–63) for radial diffusivity. To be certain that DTI values derived from an individual patient are significantly lower, with a confidence of 95%, these figures suggest that we need to have mean ROI FA values (for example) that are at least 14% lower than volunteer means. This estimate, and the secure distinction of a patient group as abnormal, is further confounded by the fact that intersubject CoV in patients with neurological disorders is larger [38], [39], and is variable across different brain regions. These figures underline the difficulty of using DTI in small groups of patients with various causes of neurological disease who have variable pathophysiology. In practice, however, estimated sample sizes in such studies are moderated by the fact that the changes in DTI are often dramatic, and significance is often detected with manageable numbers, despite the large intersubject variability in volunteer and patients groups [38], [39].

However, it is important to point out that these figures are largely irrelevant when considering the power and design of clinical studies, when DTI is being used to monitor changes within the same subject in the same scanning session (within session reproducibility) or during longitudinal assessments over time in several different imaging sessions (between session reproducibility). In such settings, the subject is his or her own control, and the relevant parameter is intrasubject variability or reproducibility. Our data show that these figures for CoV are much smaller than those obtained from the discussion in the previous paragraph. In addition, we provide reference data for FA, ADC, AD and RD in healthy volunteers demonstrating that the CoV for within session reproducibility is lower than between session reproducibility (Tables 3–6). These data provide helpful guidance for designing clinical studies, and suggest that it should be possible to detect differences of approximately 5 to 10% with confidence, particularly within single session interventional studies. For example, although the reproducibility of measurements is variable for the different brain regions we can use these data to calculate sample sizes for interventional and longitudinal clinical studies. Even when we consider the brain region with the highest CoV (ventral midbrain) we should be able to detect a 10% change in DTI with 95% power at a significance level of 1% within a group of 10 subjects within a single interventional or longitudinal study design [40]. Clearly, such estimates only strictly apply to our scanner and institution, but they provide a useful starting point for study design. There are a number of factors particular to our scanning protocols and institutional setup that limit the use of the reproducibility measurements that we provide. These include, but might not be limited to, scanner, acquisition protocols, data correction and reconstruction, and processing. Despite these variations, it should be possible for other groups to use the methodology that we describe to derive ‘in house’ data for their studies. In addition, although these data provide guidance for designing clinical studies, particular groups of subjects (including those with brain injury) may require sedation and control of ventilation as part of clinical care. While such patient groups may appear complex and difficult to manage within the context of an imaging study the fact that they remain completely immobile and have stable physiology should result in lower CoV for reproducibility measurements and an increase in the sensitivity of interventional studies [41].

### Methodological limitations

While we were able to obtain multiple DTI datasets on up to two occasions in this group of volunteers, scanner availability and subject tolerance prevented us from acquiring further DTI datasets within the same session and additional scanning sessions. We found that the within session reproducibility measurements were lower than between session reproducibility measurements obtained over a six month period. The expected change in DTI in healthy volunteers of a similar age over a period of up to six months is small and unlikely to have resulted in the differences we have found [42], [43]. The 95% prediction intervals for zero change for the within session DTI measurements were similar to that calculated from the DTI measurements obtained within all sessions. The lack of difference between these measures could be related to the fact that we were only able to obtain two sets of DTI within each session and that the 95% prediction interval for zero change for within session measurements is based on 4.3 rather than 2 SDs. These overall prediction intervals for zero change are calculated from all the ROI data, but can easily be calculated for individual ROIs using the same technique and used as a method for determining the significance of changes following an intervention or longitudinal change over time.

There were differences in the intersubject variability and reproducibility of DTI across the different brain regions. These differences are demonstrated in Tables 1–6 and figure 2, and are particularly relevant within the corpus callosum, caudate, cingulum and midbrain structures. The increase in variability and lower reproducibility of these regions may be related to partial volume errors within these relatively small structures secondary to variation in the quality of coregistration and spatial normalisation within individual subjects. We tried to limit these errors by eroding the ROI template by a single voxel to improve accuracy. Despite this, errors remain within some ROIs where DTI values differ in closely adjacent brain regions. However, the purpose of this study was to determine the variability of measurements using an ROI template and standard processing pipeline. While variability in the fitting of template ROIs in individual subjects may result in higher intersubject variability for particular brain regions this is less likely for measurements of reproducibility within the same subject. Here any differences in ROI template fitting between the sessions are likely to be small. However, these regional differences underline that DTI studies seeking to compare different subject groups or assess interventional or longitudinal change should compare data from within the same brain region using the same data processing technique. While the data we report are specific to our methods the reproducibility measurements that we report provide a useful starting point for study design.

## Conclusions

This study provides additional reference data concerning intersubject variability and reproducibility of DTI conducted in a group of healthy volunteers. The CoV for repeat DTI measurements obtained during the same session were lower than those obtained from measurements obtained in a different imaging session separated by up to six months. These data can be used to calculate the 95% prediction interval for zero change and may inform the design of interventional studies to quantify change within a single imaging session, or to assess the significance of change in longitudinal studies.
